# Clinical characteristics and genetic backgrounds of Japanese patients with atypical hemolytic uremic syndrome

**DOI:** 10.1007/s10157-018-1549-3

**Published:** 2018-03-06

**Authors:** Madoka Fujisawa, Hideki Kato, Yoko Yoshida, Tomoko Usui, Munenori Takata, Mika Fujimoto, Hideo Wada, Yumiko Uchida, Koichi Kokame, Masanori Matsumoto, Yoshihiro Fujimura, Toshiyuki Miyata, Masaomi Nangaku

**Affiliations:** 1 Division of Nephrology and Endocrinology, The University of Tokyo, 7-3-1 Hongo, Bunkyo-ku, Tokyo, 113-8655 Japan; 2 Clinical Research Support Center (CresCent), The University of Tokyo, Tokyo, Japan; 3 Department of Cardiology and Nephrology, Mie University Graduate School of Medicine, Mie, Japan; 4 Department of Molecular and Laboratory Medicine, Mie University Graduate School of Medicine, Tsu, Mie Japan; 5 Department of Molecular Pathogenesis, National Cerebral and Cardiovascular Center, Suita, Japan; 6 Department of Blood Transfusion Medicine, Nara Medical University, Nara, Japan; 7 Japanese Red Cross Kinki Block Blood Center, Ibaraki, Japan; 8 Department of Cerebrovascular Medicine, National Cerebral and Cardiovascular Center, Suita, Japan

**Keywords:** Atypical hemolytic uremic syndrome, Thrombotic microangiopathy, Congenital disorder, Complement, Epidemiology

## Abstract

**Background:**

Atypical hemolytic uremic syndrome (aHUS) is caused by complement overactivation, and its presentation and prognosis differ according to the underlying molecular defects. The aim of this study was to characterize the genetic backgrounds of aHUS patients in Japan and to elucidate the associations between their genetic backgrounds, clinical findings, and outcomes.

**Methods:**

We conducted a nationwide epidemiological survey of clinically diagnosed aHUS patients and examined 118 patients enrolled from 1998 to 2016 in Japan. We screened variants of seven genes related to complement and coagulation, as well as positivity for anti-CFH antibodies, and assessed clinical manifestations, laboratory findings, and clinical course.

**Results:**

The most frequent genetic abnormalities were in *C3* (31%) and the frequency of *CFH* variants was relatively low (10%) compared to Western countries. The predominant variant in this cohort was *C3 p.I1157T* (23%), which was related to favorable outcomes despite frequent relapses. A total of 72% of patients received plasma therapy, while 42% were treated with eculizumab. The prognosis of Japanese aHUS patients was relatively favorable, with a total mortality rate of 5.4% and a renal mortality rate of 15%.

**Conclusions:**

The common occurrence of genotype *C3*, especially the *p.I1157T* variant was the characteristic of the genetic backgrounds of Japanese aHUS patients that differed from those of Caucasian patients. In addition, the favorable prognosis of patients with the unique *C3 p.I1157T* variant indicates that understanding the clinical characteristics of individual gene alterations is important for predicting prognosis and determining therapeutic strategies in aHUS.

**Electronic supplementary material:**

The online version of this article (10.1007/s10157-018-1549-3) contains supplementary material, which is available to authorized users.

## Introduction

Atypical hemolytic uremic syndrome (aHUS) is a severe systemic disease characterized by thrombocytopenia, hemolytic anemia, and acute renal injury, and is induced by complement overactivation in the alternative pathway [1]. Variants in the genes encoding complement factor H (CFH), complement factor I (CFI), complement C3, complement factor B (CFB), membrane cofactor protein (MCP), thrombomodulin (THBD), and diacylglycerol kinase ε (*DGKE*) have been identified as inherited defects, and the production of antagonistic anti-CFH antibodies is a known acquired complement abnormality causing dysregulation of the complement pathway.

The pathogenesis of aHUS has been elucidated both by recent advances in genetic research and epidemiological studies in aHUS patients. The development of aHUS is considered to require the involvement of various environmental factors besides the underlying complement overactivation [2]. Furthermore, the clinical presentation and prognosis of aHUS differ depending on the underlying molecular abnormality. Therefore, genotype identification is clinically significant not only for aHUS diagnosis, but also for predicting each patient’s treatment response and prognosis.

Racial and regional differences were suggested by our previous report, which showed a high incidence of *C3* variants in Japanese aHUS patients in a small cohort [3, 4]. This finding suggests that aHUS patients in Japan have unique genetic backgrounds, though larger numbers of patients should be analyzed. Moreover, the influence of regional and racial differences on clinical characteristics is unclear and requires further study.

Against this background, this study aimed to document the genetic backgrounds and comprehensive characteristics of Japanese patients with initial onset aHUS, constituting the first large-scale cohort in East Asia. We clarified the racial differences in the genetic backgrounds of aHUS patients and the importance of understanding the clinical differences among individuals with various underlying abnormalities.

## Materials and methods

### Patients

We enrolled Japanese patients clinically diagnosed with aHUS from 1998 to 2016 as part of a research project with the support of the Ministry of Health, Labour, and Welfare of Japan. Diagnoses were based on Japanese guidelines for aHUS [5] (see Online Resources). Clinical and laboratory data were retrospectively collected from consultation letters or questionnaires. ESRD was defined as severe renal impairment requiring renal replacement therapy (RRT). Remission was defined as hematological remission (platelet counts above 10 × 10^4^/μl and lactate dehydrogenase (LDH) levels below 222 U/l) with preserved renal function (not reaching ESRD).

### Genetic analysis and variant interpretation

Genetic analysis was performed at the Department of Molecular Pathogenesis of the National Cerebral and Cardiovascular Center, as previously described [6, 7] (see Online Resources). Variants with amino acid substitutions in exon sequences, whose minor allele frequency (MAF) scores were < 0.005 in international databases (see Online Resources), were designated as candidate aHUS-predisposing variants.

### Autoantibodies against CFH

The presence of anti-CFH autoantibodies in each patient’s plasma or serum was evaluated using CFH IgG ELISA kits (Abnova, Taipei, Taiwan) according to the manufacturer’s instructions. Positivity was also confirmed by Western blot analysis, as previously described [6].

### Statistical analysis

Statistical analysis was performed with JMP® Pro 11.2 (SAS Institute Inc., Cary, NC, USA). Results are expressed as numerical values and median (range) for continuous variables and as percentages for categorical variables. Differences in clinical presentations and laboratory data among patients with *C3 p.I1157T* and other *C3* variants were analyzed by chi-squared or Fisher tests. Cumulative renal survival rates were estimated by Kaplan–Meier analyses, excluding dead patients, and the log-rank test was used to compare these rates between *C3 p.I1157T* and other *C3* variants. Differences were considered statistically significant at *p* < 0.05.

### Ethics statement

This study was approved by the ethical committee of The University of Tokyo Hospital (IRB G10029) and registered to UMIN-CTR (UMIN000014869). It was performed in accordance with the 1964 Declaration of Helsinki and its later amendments, and the Ethical Guidelines on Clinical Studies of the Ministry of Health, Labour and Welfare of Japan. Written informed consent was obtained from all patients or from their parents.

## Results

### Demographic backgrounds

One hundred eighteen Japanese patients from 103 families were clinically diagnosed with aHUS from 1998 to 2016 (Table 1). Initial onset occurred at a median age of 6 years, ranging from 3 months to 84 years, and during childhood (< 18 years of age) in 65% of cases. Seventy-six patients were males (64%), and this male predominance existed regardless of age (52 males and 25 females in childhood, and 24 males and 17 females in adulthood).

**Table 1:** Demographics of aHUS patients in Japan and clinical and biological characteristics at first onset: stratification by complement abnormalities

Characteristics	Overall	Genetic abnormalities				Anti-CFH Abs	Unidentified	Unanalyzed
		*C3*	*CFH*	*MCP*	*DGKE*			
Patients (*n*)	118	32	10	5	1	20	36	14
Age at initial onset (years)	6.0 (0.3–84.0)	6.0 (0.3–70.0)	26.5 (0.3–75.0)	6.0 (1.0–50.0)	0.3	6.0 (4.0–75.0)	15.0 (0.3–80.0)	4.0 (0.5–84.0)
Children (< 18 years of age) (%)	65	66	20	80	100	80	56	93
Male (%)	64	66	60	100	100	60	64	57
Family history of HUS (%)	25	47	10	40	0	0	3	79
Clinical characteristics								
Probable trigger events (%)	75	83	67	100	100	72	74	75
Gastrointestinal infections	21	17	33	0	0	28	21	0
Respiratory infections	20	17	0	0	0	39	18	25
Influenza	5	0	11	100	0	0	0	25
Other infections	13	25	11	0	100	6	9	25
Others	16	25	11	0	0	0	26	0
Duration between onset and first visit (days)	5 (0–45)	3 (0–8)	4 (0–30)	3	3	6 (1–30)	7 (0–45)	1 (0–2)
Physiological manifestations (%)								
Diarrhea	23	17	33	50	0	25	24	0
Bloody stool	6	0	11	0	0	0	12	0
Nausea or vomiting	47	17	67	50	100	60	44	40
Fever	52	42	44	100	100	40	59	60
Central nervous system manifestations	24	8	33	100	0	32	21	20
Purpura	24	8	11	50	0	40	21	40
Macrohematuria	18	42	0	50	0	25	3	60
Oligo-anuria	37	33	44	0	0	10	56	40
Serological evaluation								
Hb (g/dl)	7.4 (3.2–14.5)	8.1 (5.3–12.7)	7.2 (5.0–14.5)	10.2 (10.0–10.3)	8.9	6.4 (4.2–13.5)	7.6 (3.2–13.2)	9.1 (7.0–11.0)
Hb < 10 g/dl (%)	75	62	75	0	100	89	75	75
Hb < 6 g/dl (%)	18	8	38	0	0	16	22	0
Plt (× 10^4^/μl)	3.4 (0.2– 24.4)	3.9 (1.3–9.8)	3.0 (1.2–7.4)	4.7 (2.0–7.3)	4.1	2.6 (0.2–11.5)	4.0 (0.8–24.4)	4.1 (1.1–8.5)
Plt < 15 × 10^4^/μl (%)	97	100	100	100	100	100	94	100
Plt < 3 × 10^4^/μl (%)	42	46	50	50	0	58	28	50
LDH (U/l)	2051 (276–8800)	3069 (1165–6290)	2009 (1261–3940)	2156 (428–3884)	1830	2983 (299–8800)	1470 (392–4511)	2323 (276–7966)
Increased LDH (%)	100	100	100	100	100	100	100	100
Renal function								
eGFR (ml/min per 1.73 m^2^)	19.5 (2.2–105.3)	13.0 (7.5–62.6)	14.5 (2.7–25.1)	24.0 (2.2–45.8)	13.4	31.5 (8.6–56.9)	16.8 (4.0–105.3)	50.7 (21.1–69.8)
BUN (mg/dl)	60.0 (11.7–174.0)	54.0 (24.3–134.0)	56.0 (31.2–121.0)	71.9 (41.0–102.7)	38.8	64.8 (28.0–136.7)	60.5 (11.7–174.0)	38.5 (30.0–54.4)
AKI (%)	99	100	100	100	100	100	97	100
AKI requiring RRT (%)	48	44	70	67	100	26	55	50
Complement evaluation								
C3 (mg/dl)	74 (14–145)	68 (42–99)	87 (35–114)	79 (46–111)	20	48 (14–121)	93 (28–145)	56 (16–95)
Reduced C3 (%)	47	56	25	50	100	89	23	50
C4 (mg/dl)	23 (8–45)	28 (10–38)	29 (8–45)	23 (17–28)	14	25 (9–37)	22 (10–44)	31 (19–44)
Reduced C4 (%)	8	14	13	0	0	6	7	0
Urinary test								
Positive occult blood (%)	98	100	100	NA	NA	100	95	100
Hematuria (%)	85	100	100	NA	NA	86	78	NA
Positive proteinuria (%)	98	100	100	NA	NA	100	96	100
Urinary protein (g/gCre)	11.7 (0.6–36.3)	11.6 (8.2–14.5)	13.7 (3.9–35.9)	NA	NA	11.5 (4.1–36.3)	13.0 (0.6–28.5)	NA
Nephrotic syndrome (%)	44	50	60	NA	NA	13	55	0

Results are expressed as numerical values, medians (range) for continuous variables, and percentages for categorical variables unless otherwise indicated. Increased LDH is defined as LDH > 222 U/l. Reduced C3 and C4 are defined as < 73 and < 11 mg/dl, respectively

*HUS* hemolytic uremic syndrome, *Abs* antibodies, *Hb* hemoglobin, *Plt* platelets, *LDH* lactate dehydrogenase, *eGFR* estimated glomerular filtration rate, *BUN* blood urea nitrogen, *AKI* acute kidney injury, *RRT* renal replacement therapy, *Cre* creatinine, *NA* not available

### Clinical manifestations at the initial onset of aHUS

Clinical manifestations of each patient were evaluated at their first visit for aHUS symptoms (Table 1, left column). Overall, 75% of patients experienced possible triggering events before hospitalization. Among patients whose laboratory data were available, 25% did not manifest all three triads of thrombotic microangiopathy. Anemia (Hb levels < 10 g/dl) was observed in 75% of patients and severe anemia (< 6 g/dl) was seen in 18%. Increased levels of LDH (> 222 U/l) were found in all patients, but they ranged widely, from 276 to 8800 U/l. Thrombocytopenia (platelet count < 15 × 10^4^/μl) occurred in 97% of patients, and was severe (< 3 × 10^4^/l) in as many as 42%. All patients demonstrate acute kidney injury (except three already diagnosed with ESRD), and 48% required RRT during hospitalization. Decreased levels of C3 (< 73 mg/dl) were found in 47% of patients. Most patients demonstrated severe proteinuria and hematuria. The median proteinuria level was 11.7 g/g Cre, and 44% of patients developed nephrotic syndrome. Moreover, 98% of patients showed dipstick-positive hematuria, which was confirmed in 85% of cases by microscopic evaluation.

**Fig. 1:**
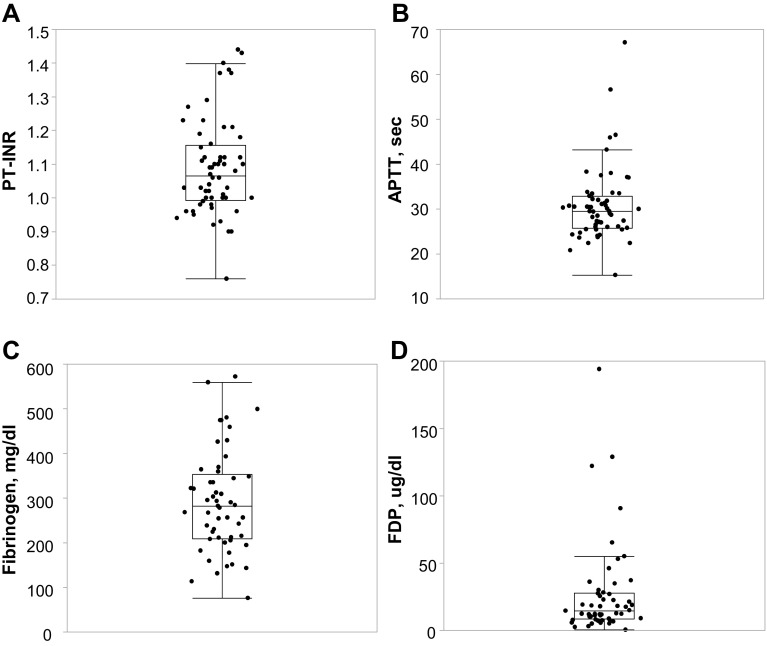
Distribution of parameters regarding coagulation and fibrinolytic factors of aHUS patients at the initial visit. Each level is expressed as median (range). **a** PT-INR (*n* = 60): 1.07 (0.76–1.44); eight patients with PT-INR > 1.25. **b** APTT (*n* = 59): 29.5 (15.3–67.1). **c** Fibrinogen (*n* = 53): 282 (76–572); five patients with fibrinogen < 150 mg/dl. **d** FDP (*n* = 53): 14.6 (0.5–194.0); 15 patients with FDP > 10 μg/dl. *PT-INR* international normalized ratio of prothrombin time, *APTT* activated partial thromboplastin time, *FDP* fibrinogen/fibrin degradation products

The coagulation–fibrinolysis system was evaluated based on the Japanese criteria for diagnosis of disseminated intravascular coagulopathy (DIC) [8] (Fig. 1). We found that 72% of patients showed increased levels of fibrinogen/fibrin degradation products (FDP) (> 10 mg/dl). However, at their initial presentation, only 13% of patients had a mildly elevated international normalized ratio of prothrombin time (PT-INR) (> 1.25), 8% demonstrated mildly decreased fibrinogen levels (from 100 to 150 mg/dl), and 2% exhibited severely decreased fibrinogen (< 100 mg/dl).

### Genetic and acquired abnormalities in patients with aHUS in Japan

We screened seven genes and anti-CFH autoantibodies, all related to the development of aHUS, in 104 patients, whose samples were available. Twenty-seven variants were detected in 48 patients (46%) (Table 2). Anti-CFH autoantibodies were identified in 20 patients, none of whom demonstrated pathogenic or rare gene variants in the seven analyzed genes. Patients were classified into the following six groups according to the identified abnormalities: C3, 32 of 104 patients (31%); CFH, 10 (10%); MCP, 5 (5%); DGKE, 1 (1%); anti-CFH autoantibody, 20 (19%); and unidentified, 36 (35%). No rare, non-synonymous variants in *CFB* or *CFI* were detected.

**Table 2:** Summary of 27 variants identified in patients with aHUS

Gene	Patient code	Inheritance	SNP rs	Nucleotide	Predicted consequence	MAF score	
						HGVD	1000 Genomes_phase3
C3	TC32	Heterozygous	–	c.535T>C	p.S179P	–	–
	TC28	Heterozygous	–	c.544T>C	p.S182P	–	–
	TC4	Heterozygous	–	c.640C>T	p.P214S	–	–
	2H	Heterozygous	rs200967589	c.1273C>T	p.R425C	0.0022	0.0006
	TC3	Heterozygous	rs767334972	c.1663G>A	p.V555I	–	–
	TC42	Heterozygous	–	c.3125G>T	p.R1042L	–	–
	3O	Heterozygous	–	c.3313A>C	p.K1105Q	–	–
	F1, F2, F3, F5, G1, G2, G3, H1, H2, 2A, 2J1, 2J2, 2N, 2Q, 2S, 2U, 2V, 2Z, 3M, 3V, 4B, 4G1, 4G2, TC46	Heterozygous	–	c.3470T>C	p.I1157T	–	–
	3Z	Heterozygous	–	c.3478G>A	p.E1160K	–	–
CFH	TC20^a^	Heterozygous	rs762132970	c.526T>C	p.F176L	–	–
	TC36	Heterozygous	–	c.695G>A	p.R232Q	–	–
	TC14^a^	Heterozygous	–	c.1951C>T	p.H651Y	–	–
	TC7	Heterozygous	rs55931547	c.2392G>A	p.D798N	0.002	–
	TC33	Heterozygous	–	c.3572C>G	p.S1191W	–	–
	TC14	Heterozygous	–	c.3593A>T	p.E1198V	–	–
	TC1	Heterozygous	–	c.3594A>T	p.E1198D	–	–
	2M, TC8	Heterozygous	rs121913051	c.3643C>G	p.R1215G	–	–
	X, 2I, TC20	Heterozygous	–	c.3644G>A	p.R1215Q	–	–
THBD	2I^a^	Heterozygous	–	c.1499C>T	T500M	–	–
MCP	M1, M2	Homozygous	–	c.191G>A	p.C64Y	–	–
	F3^a^, F5^a^, TC43	Heterozygous	rs116800126	c.293C>T	p.T98I	0.0036	0.001
	P	Homozygous	–	c.509delA	p.N170Mfs^a^9	–	–
	2D	Heterozygous	rs202071781	c.565T>G	p.Y189D	0.000008	0.0002
	2U^a^	Heterozygous	rs773860894	c.583C>T	p.P195S	0.00002	–
	2D^a^	Heterozygous	rs767322836	c.1076C>T	p.A359V	0.00002	–
DGKE	2R	Heterozygous	–	c.71delT	p.L24Cfs^a^145	–	–
	2R	Heterozygous	–	c.1213-2A>G	r.spl? p.(A405_E428del)	–	–

*MAF* minor allele frequency

^a^Patients who had another candidate aHUS-predisposing variant with known pathogenicity

Genetic testing revealed that compared to Western countries, the frequency of *CFH* variants in Japan (10%) was lower [2, 9] while that of *C3* variants was higher (31%). Of note, the *C3 p.I1157T* variant was detected in 24 patients (23%) from 16 families.

Six patients had combined mutations: three carrying *C3 p.I1157T* also had heterozygous *MCP* variants with unknown pathogenicity; two from the same family had *MCP p.T98I*, and one had *MCP p.P195S*. One patient had *THBD p.T500M* in addition to *CFH p.R1215Q*, the latter of which is located in a mutational hotspot region of CFH and was reported to impair function [10]. One patient had a combination of two heterozygous *MCP* variants, *p.Y189D* and *p.A359V*, the first of which was shown to be predisposing [11]. We screened for *DGKE* variants in 22 patients under 2 years [12, 13] and identified one patient (5%) with compound heterozygous variants [7, 14].

### Clinical characteristics according to complement abnormalities

We then compared the clinical characteristics of aHUS patients according to genetic or acquired backgrounds. Age at the initial aHUS onset differed depending on the underlying abnormality, but the initial episode usually occurred during childhood in the C3, MCP, and unidentified groups (Table 1 and Online Resource 1). Patients with anti-CFH antibodies showed a biphasic distribution of age at aHUS onset: during childhood (from ages 4 to 11) in 80% and during middle-to-old age in the remaining 20%. Males were more commonly affected than females in all groups. A family history of aHUS was frequent in the C3 (47%) and MCP (40%) groups, was rare in the CFH (10%) and the unidentified (3%) groups, and was absent in the DGKE and anti-CFH antibody groups. Although most laboratory data at the first visit after initial aHUS onset did not differ between groups, severe anemia was observed in patients with *CFH* variants (38%), and the ratio of patients with reduced C3 levels varied by abnormality. Approximately half of the patients with *C3, MCP* variants, one patient with *DGKE* variant, and 89% of the patients with anti-CFH antibody showed decreased levels of C3.

### Treatment at the initial onset of aHUS and subsequent outcomes

Information on treatment administered at the initial onset of aHUS was available in 101 patients (Table 3). Seventy-four patients were treated with plasma therapy at a median of 2 days (1–38 days) after admission, including 33 who began taking eculizumab at a median of 10 days (1–181 days) after starting plasma therapy. One patient was treated with eculizumab only. Fifty-two patients were diagnosed with aHUS after eculizumab was approved for the treatment of aHUS in Japan in September 2013. Thereafter, the overall treatment approach to aHUS changed and 54% (28/52) of patients were treated with eculizumab.

**Table 3:** Treatment and outcome of patients with aHUS according to each complement abnormality

Patients	Overall	Genetic abnormalities				Anti-CFH Abs	Unidentified	Unanalyzed
		*C3*	*CFH*	*MCP*	*DGKE*			
Patients (*n*)	118	32	10	5	1	20	36	14
Follow-up^a^ (years)	2.3 (0–54.0)	17.0 (0.1–54.0)	1.6 (0.5–9.0)	11.0 (0.3–29.0)	1.5	2.0 (0–25.0)	1.0 (0–6.0)	1.5 (0–37.0)
Treatment (*n*)								
At initial onset: Ob/PT/ECZ ± PT^b^	26/41/34	12/10/3	1/3/6	1/3/0	0/0/1	0/10/9	9/10/15	3/5/0
Before approval of ECZ	20/23/6	11/7/0	1/2/0	1/2/0	0/0/1	0/6/2	4/3/3	3/3/0
After approval of ECZ	6/18/28	1/3/3	0/1/6	0/1/0	–	0/4/7	5/7/12	0/2/0
Current: non-use/use of ECZ	81/27	26/6	3/7	5/0	0/1	14^d^/4	22/9	11/0
Outcome at discharge after initial onset, *n* (%)								
Remission	95 (84)	30 (94)	6 (60)	5 (100)	1 (100)	17 (89)	25 (71)	11 (100)
ESRD	14 (12)	1 (3)	3 (30)	0 (0)	0 (0)	2 (11)	8 (23)	0 (0)
Death	4 (4)	1 (3)	1 (10)	0 (0)	0 (0)	0 (0)	2 (6)	0 (0)
Long-term outcome^a^								
Relapse (%)	38	77	33	50	100	0	4	73
Number of relapses	0 (0–9)	2 (0–9)	0 (0–2)	0 (0–4)	1	0	0 (0–1)	2 (0–5)
Relapse within a year of initial onset (%)	36	23	100	0	–	–	0	60
Time until the second onset^c^ (years)	3.0 (0.3–13.0)	3.0 (0.4–13.0)	0.3	7.0 (5.0–9.0)	NA	–	NA	1.0 (0.5–13.0)
Treatment for relapses: Ob/PT/ECZ ± PT^b^	6/12/8	5/8/5	0/1/2	0/2/0	0/0/1	–	0/1/0	1/2/0
Outcome reported on latest questionnaire, n (%)								
Remission	94 (83)	26 (81)	7 (70)	5 (100)	1 (100)	19 (100)	25 (71)	11 (100)
ESRD	13 (12)	5 (16)	2 (20)	0 (0)	0 (0)	0 (0)	6 (17)	0 (0)
Death	6 (5)	1 (3)	1 (10)	0 (0)	0 (0)	0 (0)	4 (11)	0 (0)

Results are expressed as numerical values, median (range) for continuous variables and percentages for categorical variables unless otherwise indicated

*Ob* observational therapy, *PT* plasma therapy (plasma infusion or plasma exchange), *ECZ* eculizumab, *ESRD* end-stage renal disease (requiring renal replacement therapy), *NA* not available

^a^Long-term outcome was based on the latest questionnaire

^b^Patients categorized in the ECZ group were treated with eculizumab with or without plasma therapy

^c^Relapses after transplantation are not included

^d^Eight out of 13 patients were receiving immunosuppressive therapy

The mortality rate of aHUS patients in the acute phase was 3.3% and did not differ according to abnormality. The overall renal mortality rate at discharge was 12.8%, but was higher in *CFH* variants (38%) and the unidentified group (24%). The risk of death or ESRD in the acute phase did not differ by treatment choice for any abnormality (Online Resource 2).

### Long-term outcomes

**Fig. 2:**
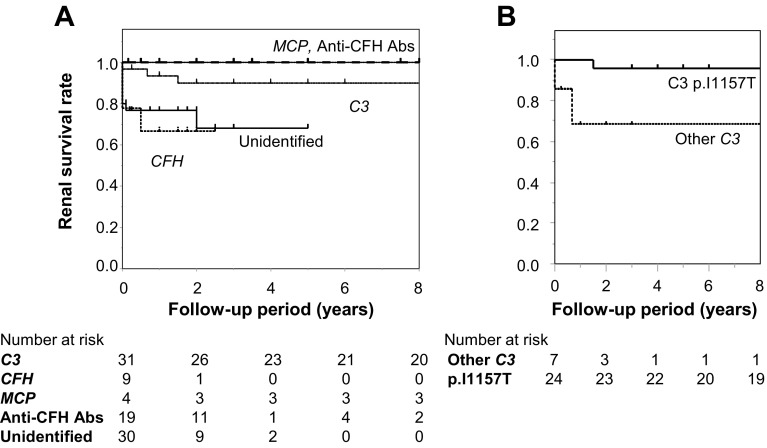
Cumulative renal survival of aHUS patients according to each abnormality estimated by Kaplan–Meier analyses. **a** Patients with *MCP* variants and anti-CFH antibodies had never reached end-stage renal disease. Patients with *C3* variants had good renal outcomes, in contrast with patients with *CFH* variants and patients in the unidentified group. **b** Among patients with *C3* variants, patients with the *C3 p.I1157T* variant had better renal survival than patients with other *C3* variants (log-rank test, *p* = 0.0003). *Anti-CFH Abs* anti-CFH antibodies

Patients were followed for a median of 2.5 years (1 month to 54 years) after the initial onset of aHUS, both retrospectively and prospectively in 104 patients. The total mortality rate was 5.4%, with two patients dying within a year after hospital discharge (Table 3 and Online Resource 3), and the renal mortality rate was 15% (Fig. 2 and Online Resource 4). Relapses occurred in patients with *DGKE* (100%), *C3* (77%), *MCP* (50%), and *CFH* (38%) variants.

Forty-one patients received eculizumab therapy, including those treated for relapses. Eculizumab was discontinued in 13 patients, 12 of whom maintained remission for a median of 1 year (0.5–3 years), while one patient with *C3 p.I1157T* relapsed and resumed eculizumab treatment [15] (Online Resource 5). All 27 patients who received eculizumab as maintenance therapy were free from relapses, ESRD, and death, except for the single patient with a *DGKE* variant.

Among the 20 patients with anti-CFH antibodies, 8 were treated with immunosuppressive medication, 5 with observational therapy, and 4 with eculizumab, each as maintenance therapy. Two patients were able to withdraw from RRT (Online Resource 4) more than a year after aHUS onset, and ultimately all 20 patients were free from relapse, ESRD, and death.

Renal transplantation was performed in three patients, all of whom experienced aHUS relapse after transplantation. One with *C3* variant redeveloped ESRD, while the other two, one with CFH variant and the other in the unidentified group, recovered renal function (Online Resource 4).

### *C3 p.I1157T* variant compared to other *C3* variants

The clinical characteristics of patients with the *C3 p.I1157T* variant differed in several ways from those of patients with other *C3* variants (Table 4). Interestingly, reduced C3 levels at aHUS onset were observed in none of the patients with *C3 p.I1157T* but all of those with other *C3* variants (Table 4). The median number of relapse episodes in patients with *C3 p.I1157T* was significantly higher than in those with other *C3* variants (2 vs. 0, *p* = 0.016). However, patients carrying *C3 p.I1157T* showed better outcomes, both in the acute phase (*p* = 0.041, Table 4) and during long-time follow-up (*p* = 0.023). Furthermore, many *C3 p.I1157T* patients achieved remission with only supportive care (65%) or plasma therapy (35%).

**Table 4:** Comparison of patients with *C3 p.I1157T* and other *C3* variants

Patients with C3 variants	p.I1157T	Other variants	*p* value
Patients (*n*)	24	8	
Age at initial onset (years)	6.0 (1.0–70.0)	5.4 (0.3–48.0)	0.57
Children (< 18 years of age) (%)	67	63	0.99
Male (%)	71	50	0.40
Family history of aHUS (%)	58	13	0.041
Follow-up^a^ (years)	21.0 (3.0–54.0)	2.3 (0.1–35.0)	0.012
Clinical presentation (%)			
Anemia			
Hb < 10 g/dl	29	100	0.021
Hb < 6 g/dl	0	17	0.46
Thrombocytopenia			
Plt < 15 × 10^9^/μl	100	100	
Plt < 3 × 10^9^/μl	57	33	0.59
AKI	100	100	
AKI requiring RRT	41	50	0.99
Reduced C3	0	100	0.008
Treatment			
At initial onset			
Ob/PT/ECZ^b^	11/6/0	1/4/3	0.008
Before approval of ECZ^b^	10/5/0	1/2/0	
After approval of ECZ^b^	1/1/0	0/2/3	
Current			
Non-use/use of ECZ	21/3	5/3	0.12
Outcome at discharge after initial onset, *n* (%)			0.041
Remission	24 (100)	6 (75)	
ESRD	0 (0)	1 (13)	
Death	0 (0)	1 (13)	
Long-term outcome^a^			
Relapse (%)	88	33	0.016
Number of relapses	2 (0–9)	0 (0–3)	0.003
Relapse within a year of initial onset (%)	25	0	0.99
Time until the second onset^c^ (years)	3.0 (0.4–13.0)	4.0	0.38
Treatment for relapses: Ob/PT/ECZ ± PT^b^	5/7/5	0/1/0	0.86
Outcome reported on latest questionnaire, *n* (%)			0.023
Remission	22 (92)	4 (50)	0.041
ESRD	2 (8)	3 (38)	0.012
Death	0 (0)	1 (13)	

Results are expressed as numerical values, median (range) for continuous variables and percentages for categorical variables unless otherwise indicated

*HUS* hemolytic uremic syndrome, *Hb* hemoglobin, *Plt* platelets, *AKI* acute kidney injury, *RRT* renal replacement therapy, *Ob* observational therapy, *PT* plasma therapy (plasma infusion or plasma exchange), *ECZ* eculizumab, *ESRD* end-stage renal disease (requiring renal replacement therapy)

^a^Long-term outcome was based on the latest questionnaire

^b^Patients categorized in the ECZ group were treated with eculizumab with or without plasma therapy

^c^Relapses after transplantation are not included

## Discussion

We report here a nationwide aHUS cohort in Japan in the first study from East Asia to include a substantial number of patients, as well as one of the largest global aHUS cohort series. We included 118 patients clinically diagnosed with aHUS over the last two decades and confirmed higher frequencies of *C3* variants and anti-CFH antibodies and lower frequencies of *CFH* variants in Japanese patients compared with Caucasians [2, 9, 16]. These findings agree with those of cohorts from East Asia with limited age distributions or small sample sizes [17, 18]. Moreover, in this study, we expanded our aHUS previous cohort [4], and reconfirmed that *C3* variants are frequent in Japan, and demonstrated that the most common genetic abnormality was *C3 p.I1157T*. This variant has not been found among East Asian and Japanese healthy populations. Thus, we revealed that genetic backgrounds differ by region and race.

Patients with the unique *C3 p.I1157T* variant were notable not only for their high frequency of relapses, but also for their favorable prognosis; 92% of them remained in remission (median and average follow-up was 21 and 22 years, respectively) despite repeated relapse episodes. Relapses could usually be treated successfully with just supportive care, and in that respect differed from relapses of patients with other *C3* variants. These findings not only reaffirm the importance of characterizing the genotype–phenotype correlation in aHUS [2, 9, 19, 20], but also suggest that clinical presentation can be influenced differently by particular variants in the same defective protein. Furthermore, this finding raises a recently debated issue [21], namely, the permanent use of eculizumab for managing aHUS. Our study of *C3 p.I1157T* indicates the importance of understanding differences in the natural history of aHUS and treatment duration and decision of discontinuation might depend on individual genetic variations.

Since there is no objective test for diagnosing aHUS except for time-consuming genetic analysis, diagnosis depends on recording clinical manifestations and basic laboratory data at the first visit, especially for initial disease onset. The following three findings provide additional clues supporting its clinical diagnosis. First, nearly, half of aHUS patients, regardless of their underlying pathogenic abnormalities, presented with severe thrombocytopenia (< 3 × 10^4^/μl). While this condition is a well-known characteristic of TTP in thrombotic microangiopathy [22], our data suggest that it may not only indicate TTP, but also aHUS. Second, many patients exhibited severe hematuria and proteinuria. Although those with *DGKE* variants are known to manifest nephrotic syndrome [12], we observed severe proteinuria in up to 50% of patients with other genetic abnormalities. Finally, aHUS patients showed activation of the coagulation and fibrinolytic systems without severe consumption of clotting factors. Even though differentiating between DIC and aHUS has been controversial [23], our laboratory data indicated that most aHUS patients do not meet the criteria for DIC.

Furthermore, we reported findings regarding practical treatments and their outcomes not only in the acute phase, but also over relatively long periods. Renal outcomes in Japanese patients were better than those in Caucasian patients [9, 24], even before eculizumab was introduced. This is probably due to the unique genetic background of the Japanese population, specifically the predominance of *C3 p.I1157T* and anti-CFH antibodies. This background also likely explains the lack of significant differences between the outcomes of patients treated with eculizumab (overall renal mortality rate: 20%) and without eculizumab (renal mortality rates of patients treated with observational therapy and plasma therapy: 13 and 9%, respectively) (*p* = 0.22, Online Resource 2). The reason we observed better outcomes in patients with anti-CFH antibodies compared to previous reports [2, 25] is unclear, but it too may be due to the unique characteristics of patients in Japan. The optimal duration of eculizumab use and possible withdrawal effects are ongoing questions in the management of patients with various aHUS abnormalities [21, 26] and our report of 13 cases of eculizumab discontinuation could provide new insight into this question.

One limitation of this study is that aHUS was not a well-understood entity in clinics approximately 20 years ago, when we started enrolling aHUS patients in Japan (Online Resource 6) [5, 27]. Thus, patients who eventually required permanent RRT might not have been enrolled in this study, which may have improved the prognosis in our cohort. Actually, the risk for ESRD and death did not change before and after the approval of eculizumab (13 and 19%, respectively). Another limitation is that this was an observational study and the prognosis could have change with the development of treatment for aHUS.

In conclusion, our study describes the genetic and clinical features of Japanese patients with aHUS, and is the first analysis of this topic in a large cohort of Asian patients. Our results suggest that the etiologies of aHUS might differ among continents and races. The *C3 p.I1157T* variant was predominant in Japan, was associated with a favorable prognosis, and differed from other previously reported *C3* variants. Our findings emphasize the importance of a detailed understanding of each patient’s genotype and its corresponding clinical characteristics, as this knowledge is necessary to help determine treatment strategies, including eculizumab discontinuation.

## Electronic supplementary material

Below is the link to the electronic supplementary material.

Supplementary material 1 (DOCX 171 KB)
