# Correction to: Clinical characteristics and genetic backgrounds of Japanese patients with atypical hemolytic uremic syndrome

**DOI:** 10.1007/s10157-019-01728-3

**Published:** 2019-04-23

**Authors:** Madoka Fujisawa, Hideki Kato, Yoko Yoshida, Tomoko Usui, Munenori Takata, Mika Fujimoto, Hideo Wada, Yumiko Uchida, Koichi Kokame, Masanori Matsumoto, Yoshihiro Fujimura, Toshiyuki Miyata, Masaomi Nangaku

**Affiliations:** 1 Division of Nephrology and Endocrinology, The University of Tokyo, 7-3-1 Hongo, Bunkyo-ku, Tokyo, 113-8655 Japan; 2 Clinical Research Support Center (CresCent), The University of Tokyo, Tokyo, Japan; 3 Department of Cardiology and Nephrology, Mie University Graduate School of Medicine, Mie, Japan; 4 Department of Molecular and Laboratory Medicine, Mie University Graduate School of Medicine, Tsu, Mie Japan; 5 Department of Molecular Pathogenesis, National Cerebral and Cardiovascular Center, Suita, Japan; 6 Department of Blood Transfusion Medicine, Nara Medical University, Nara, Japan; 7 Japanese Red Cross Kinki Block Blood Center, Ibaraki, Japan; 8 Department of Cerebrovascular Medicine, National Cerebral and Cardiovascular Center, Suita, Japan

## Correction to: Clinical and Experimental Nephrology (2018) 22:1088–1099 10.1007/s10157-018-1549-3

The article “Clinical characteristics and genetic backgrounds of Japanese patients with atypical hemolytic uremic syndrome, written by Madoka Fujisawa, Hideki Kato, Yoko Yoshida, Tomoko Usui, Munenori Takata, Mika Fujimoto, Hideo Wada, Yumiko Uchida, Koichi Kokame, Masanori Matsumoto, Yoshihiro Fujimura, Toshiyuki Miyata and Masaomi Nangaku was originally published electronically on the publisher’s internet portal (currently SpringerLink) on 6th March 2018 without open access.

With the author(s)’ decision to opt for Open Choice the copyright of the article changed on 5th April 2019 to © The Author(s) 2019 and the article is forthwith distributed under the terms of the Creative Commons Attribution 4.0 International License (http://creativecommons.org/licenses/by/4.0/), which permits use, duplication, adaptation, distribution and reproduction in any medium or format, as long as you give appropriate credit to the original author(s) and the source, provide a link to the Creative Commons license and indicate if changes were made. The original article has been corrected.

