# Supplementary material for: Clinical characteristics and genetic backgrounds of Japanese patients with atypical hemolytic uremic syndrome
Source: Clin Exp Nephrol. 2018 Mar 6;22(5):1088–99. doi: 10.1007/s10157-018-1549-3 (PMC6437120; doi:10.1007/s10157-018-1549-3)
Supplement: Supplementary file 1 — Supplementary material 1 (DOCX 171 KB) [file 10157_2018_1549_MOESM1_ESM.docx]

**Supplementary Methods**

**Patients**

Briefly, thrombotic microangiopathy (TMA) was diagnosed if patients fulfilled at least two of the following criteria: hemolytic anemia (hemoglobin [Hb] levels < 10 g/dl), thrombocytopenia (platelet [Plt] counts < 15×10^4^/ul), and acute kidney injury (AKI). Patients with TMA were clinically diagnosed with aHUS if the following diagnoses could be excluded: Shiga toxin–producing Escherichia coli (STEC)-HUS, thrombotic thrombocytopenic purpura (TTP), and secondary TMA.

Patients were enrolled from the nationwide TMA cohort of Nara Medical University from 1998 to August 2014, and from the aHUS cohort at The University of Tokyo from September 2014 to September 2016, as a research project with the support of the Ministry of Health, Labour, and Welfare of Japan. The case histories of 14 patients (1-11) and the genetic results of 41 patients were previously reported (12-15). Clinical and laboratory data obtained at the first visit for the initial onset of aHUS were retrospectively collected for each patient from the consultation letters to Nara Medical University or from the questionnaires sent from The University of Tokyo to each facility. The data were registered to UHCT ACReSS (University Hospital Clinical Trial Alliance in Japan). Entries were accepted even if some clinical data were missing. Follow-up information was updated from the latest questionnaires sent to each hospital. Patients with candidate aHUS-predisposing variants or anti-CFH antibodies were classified into each abnormality group. Patients in whom no inherited or acquired abnormalities were detected were classified into the no-abnormality group, and patients who did not receive analyses were classified into the unanalyzed group.

**Genetic analysis and variant interpretation**

The entire coding regions of the genes for CFH (NM 000186.3), CFI (NM 000204.3), MCP (NM 002389.4), CFB (NM 001710.5), C3 (NM 000064.2), THBD (NM 000361.2), and DGKE (NM 003647.2) were analyzed by direct DNA sequencing following PCR amplification. *DGKE* was analyzed only in patients under 2 years old. The adenine of the ATG translation initiation start site was numbered as +1, and the initial Met was denoted as +1.

Candidate aHUS-predisposing variants were defined as those with amino acid substitutions in exon sequences and with minor allele frequency (MAF) scores <0.005 as listed in the following international databases, searched in September 2016: the National Center for Biotechnology Information dbSNP database (www.ncbi.nlm.nih.gov/snp), the Human Genetic Variation Database (http://www.hgvd.genome.med.kyoto-u.ac.jp/), and the 1000Genomes Project phase 3 (http://browser.1000genomes.org/index.html). The *THBD p.D486Y* variant that was reported as predisposing to aHUS (16) was excluded from our candidate lists because its MAF scores were >0.005 in all databases.

**Online Resource 1. Distribution of ages at onset of aHUS according to each abnormality.**


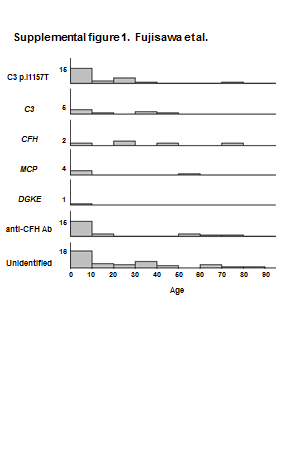


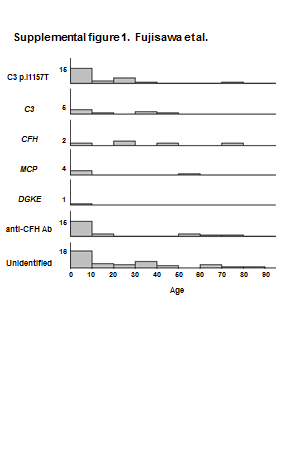


**Unidentified**

**anti-CFH Ab**

***DGKE***

***MCP***

***CFH***

***C3***

**C3 p.I1157T**

**1**

**16**

**15**

**4**

**2**

**5**

**15**

**Online Resource 1. Fujisawa et al.**


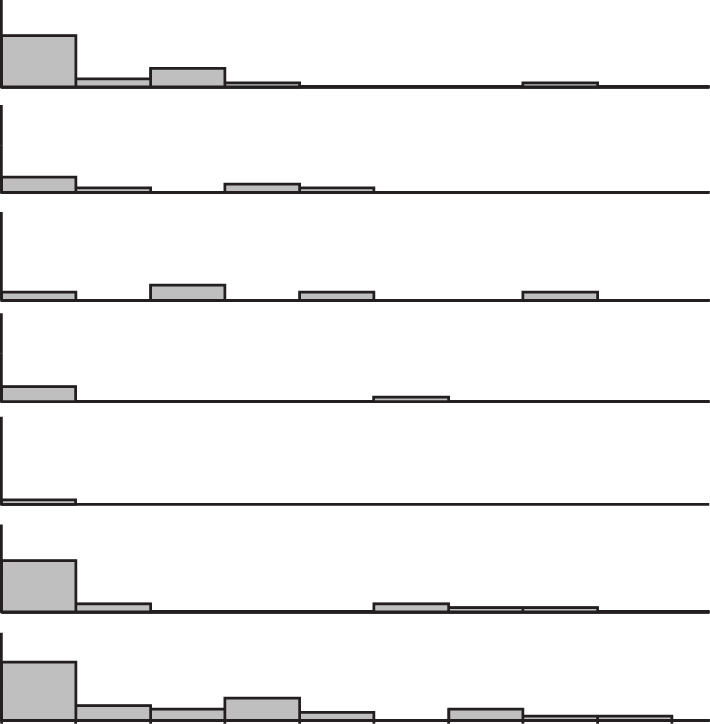


**0**

**10**

**20**

**30**

**40**

**50**

**60**

**70**

**80**

**90**

**Online Resource 2: Treatment for the initial onset of aHUS in patients who reached ESRD: stratification by complement abnormality**

| Complement abnormality | Treatment for initial onset | | | p value |
| --- | --- | --- | --- | --- |
|  | Ob | PT | ECZ±PT^a^ |  |
| **Outcome of ESRD** |  |  |  |  |
| All | 3 / 24 | 3 / 34 | 7 / 29 | 0.22 |
| *C3 p.I1157T* | 0 / 11 | 0 / 6 | 0 / 0 |  |
| Other *C3* variants | 0 / 1 | 0 / 3 | 1 / 3 | 0.46 |
| *CFH* | 1 / 1 | 0 / 2 | 2 / 6 | 0.22 |
| *MCP* | 0 / 1 | 0 / 3 | - |  |
| *DGKE* | - | - | 0 / 1 |  |
| Anti CFH-Abs | 0 / 0 | 1 / 10 | 1 / 9 | 0.99 |
| Unidentified | 2 / 8 | 2 / 10 | 4 / 14 | 0.89 |
|  |  |  |  |  |

Results are expressed as number reaching ESRD / number receiving each treatment, excluding patients who died. Abbreviations: ESRD, end-stage renal disease; Ob, observational therapy; PT, plasma therapy (plasma infusion or plasma exchange); ECZ, eculizumab.

^a^ Patients categorized into the ECZ group were treated by eculizumab with or without plasma therapy.

**Online Resource 3: Summary of patients who died for reasons related to aHUS onset.**

| Patient Code | Complement abnormality | Age at onset, years | Past history | Treatment | | Status at discharge | | | | Duration between initial visit and time of death | Cause of death |
| --- | --- | --- | --- | --- | --- | --- | --- | --- | --- | --- | --- |
| **Patients who died during hospitalization for initial aHUS onset** | | | | | | | | | | | |
| 2H | *C3 p.R425C* | 0.7 | Fallot’s syndrome | | PT | | Death | | 2 months | | Brain edema (aHUS occurred just after surgery for Fallot’s syndrome and the direct cause of death was not clear) |
| TC7 | *CFH p.D798N* | 75.0 | Rapidly progressive glomerulonephritis  (5 months before onset), DM, CHF | | PT | | Death | | 6 months | | Worsening of general status with no aggressive treatment considering patient’s old age |
| TC15 | Unidentified | 0.6 | Hutchinson-Gilford progeria syndrome | | PT, ECZ | | Death | | 3 months | | Heart failure and sick sinus syndrome due hypertension or hypervolemia |
| TC31 | Unidentified | 0.3 | Anemia and jaundice | | Ob | | Death | | 2 months | | Cardiac/respiratory arrest due to severe anemia |
| **Patients who died after initial onset** | | | | | | | | | | | |
| TC25 | Unidentified | 76.0 | None | | PT | | Serological remission, ESRD | 6 months (2 months after discharge) | | | Melena |
| TC37 | Unidentified | 80.0 | None | | PT, ECZ | | Remission | 10 months (7 months after discharge) | | | Multi-organ failure with pleurisy |

Abbreviations: DM, Diabetes mellitus; CHF, Chronic heart failure; ESRD, end-stage renal disease (requiring renal replacement therapy); Ob, observational therapy; PT, plasma therapy (plasma infusion or plasma exchange); ECZ, eculizumab.

**Online Resource 4: Clinical characteristics of all patients who reached ESRD**

| Patient Code | Complement abnormality | Age at onset, years | Number of relapses | | Treatment for initial onset | | Current treatment | | | | Current status | | | |
| --- | --- | --- | --- | --- | --- | --- | --- | --- | --- | --- | --- | --- | --- | --- |
| **Patients who reached to ESRD during the first onset** | | | | | | | | | | | | | | |
| 3H | Unidentified | 0.3 | 0 | | PT, ECZ | | ECZ | | | | ESRD | | | |
| TC45 | Unidentified | 30.0 | 0 | | PT, ECZ | | ECZ | | | | ESRD | | | |
| TC4 | *C3 p.P214S* | 32.0 | 0 | | PT, ECZ | | ECZ | | | | ESRD | | | |
| TC40 | Unidentified | 35.0 | 0 | | Ob | | none | | | | ESRD | | | |
| 3A | Unidentified | 37.0 | 0 | | PT, ECZ | | ECZ | | | | ESRD | | | |
| TC36 | *CFH p.R232Q* | 43.0 | 0 | | PT, ECZ | | none | | | | ESRD | | | |
| TC44 | Unidentified | 61.0 | 0 | | PT, ECZ | | none | | | | ESRD | | | |
| TC25 | Unidentified | 76.0 | 0 | | PT | | none | | | | Died 6 months after hospitalization for aHUS onset | | | |
| Patient Code | Complement abnormality | Age at onset, years | Number of relapses | Treatment for initial onset | | Treatment for relapses | | Time to reach ESRD | | Cause of ESRD | | | | Current status |
| **Patients who reached ESRD after remission following initial onset** | | | | | | | | | | | | | | |
| 3Z | *C3 p.E1160K* | 0.3 | 1 (after transplantation) | PT | | Posttransplantation therapy | | 0.7 | | Gradual impairment | | | | ESRD, after posttransplantation onset |
| TC41 | Unidentified | 0.4 | 0 | Ob | | - | | 2.0 | | Gradual impairment | | | | ESRD |
| 2J1 | *C3 p.I1157T* | 2.0 | 6 | PT | | PT | | 36.0 | | Gradual impairment after 6th relapse | | | | ESRD |
| TC42 | *C3 p.R1042L* | 10.0 | 3 | Ob | | PT | | 9.0 | | 4th relapse | | | | ESRD |
| 2I | *CFH p.R1215Q, THBD p.T500M* | 28.0 | 1 | PT | | PT | | 0.5 | | 1st relapse | | | | ESRD |
| 2J2 | *C3 p.I1157T* | 70.0 | 0 | N.A. | | - | | 1.5 | | Gradual impairment, partly caused by unilateral nephrectomy for renal cancer | | | | ESRD |
| Patient Code | Complement abnormality | Age at onset, years | Number of relapses | | Treatment for initial onset | Current treatment | | | Time until discontinuation of RRT, years | | Cause of renal recovery | Current status | | |
| **Patients who once reached to and recovered from ESRD** | | | | | | | | | | | | | | |
| 3L | Anti-CFH Abs | 4.0 | 0 | | PT, IMT | IMT | | | 1.0 | | Gradual recovery | | Maintaining remission 6 years for renal recovery | |
| 3Y | Anti-CFH Abs | 58.0 | 0 | | PT, ECZ | none | | | 1.3 | | Gradual recovery | | Maintaining remission 2 years for renal recovery | |
| TC33 | *CFH p.S1191W* | 24.0 | 0 | | PT, ECZ | ECZ | | | 1.5 | | Gradual recovery | | Maintaining remission 6 months for renal recovery | |
| 2F | Unidentified | 3.0 | 0 | | Ob | none | | | 2.0 | | Gradual recovery | | Maintaining remission 3 years after for recovery | |
| 2K | Unidentified | 32.0 | 1 (after transplantation) | | PT | Posttransplantation therapy | | | 2.5 | | Renal transplantation | | Maintaining remission 6 months for renal transplantation | |
| X | *CFH p.R1215Q* | 22.0 | 1 (after transplantation) | | Ob | Posttransplantation therapy, ECZ | | | 8.0 | | Renal transplantation | | Maintaining remission 5 years for renal transplantation | |

Abbreviations: ESRD, end-stage renal disease; Ob, observational therapy; PT, plasma therapy (plasma infusion or plasma exchange); IMT, immunosuppressive therapy; ECZ, eculizumab.

**Online Resource 5: Details of patients who discontinued ECZ treatment.**

| Patient Code | Complement abnormality | Age at aHUS onset, yr | ECZ initiation | ECZ duration | Status after ECZ discontinuation |
| --- | --- | --- | --- | --- | --- |
| 3M | *C3 p.I1157T* | 9.0 | At 2nd relapse | 0.4 yrs | Relapse occurred 4 months after discontinuation and ECZ was reintroduced |
| G2 | *C3 p.I1157T* | 14.0 | At 2nd relapse | 1.0 yr | Maintaining remission for 1.5 yrs |
| 3V | *C3 p.I1157T* | 3.0 | At 3rd relapse | Twice | Maintaining remission for 3.0 yrs |
| 4B | *C3 p.I1157T* | 1.0 | At 3rd relapse | Once | N.A. |
| TC36 | *CFH p.R232Q* | 43.0 | At initial onset (Status at discharge: ESRD) | 0.1 yrs | Maintaining remission for 0.5 yrs |
| W | Anti-CFH Abs | 8.0 | At initial onset | 5.0 yrs | Maintaining remission for 1 yr with the use of immunosuppressive therapy |
| 4A | Anti-CFH Abs | 5.0 | At initial onset | 1.0 yr | Maintaining remission for 1 yr with the use of immunosuppressive therapy |
| TC22 | Anti-CFH Abs | 11.0 | At initial onset | 1.5 yrs | Maintaining remission for 0.5 yrs with the use of immunosuppressive therapy |
| 3Y | Anti-CFH Abs | 58.0 | At initial onset | Once | Maintaining remission for 3.0 yrs |
| 3X | Anti-CFH Abs | 69.0 | At initial onset | Once | Maintaining remission for 2.0 yrs with the use of immunosuppressive therapy |
| TC12 | Unidentified | 65.0 | At initial onset | 1.5 yrs | Maintaining remission for 1.0 yr |
| TC29 | Unidentified | 16.0 | At initial onset | 1.5 yrs | Maintaining remission for 0.5 yrs |
| TC34 | Unidentified | 62.0 | At initial onset | 0.2 yrs | Maintaining remission for 1.3 yrs |

Abbreviations: ESRD, end-stage renal disease; yr, year

**Online Resource 6. The number of patients who was consulted to our institutions in each year of the enrollment period.**


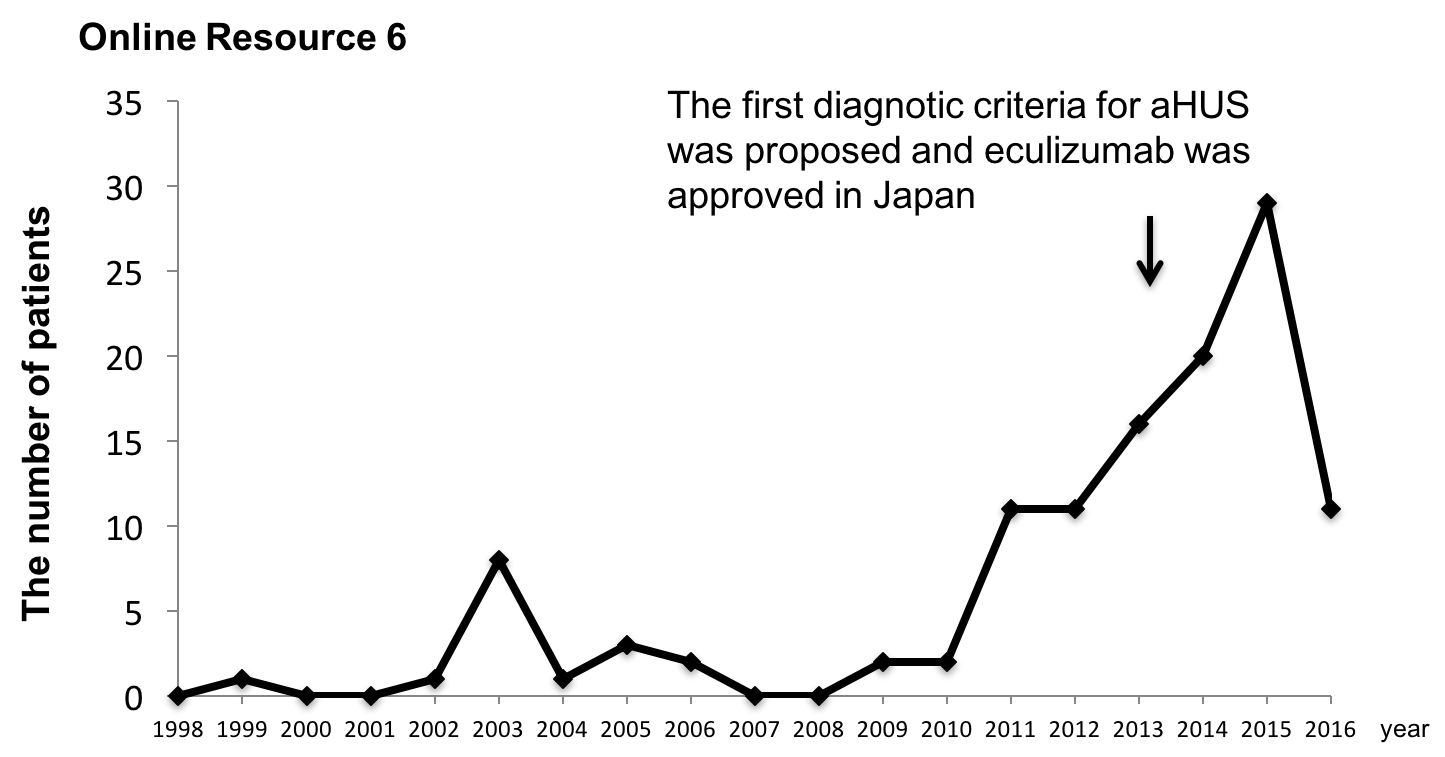


The number increased around the year 2013, when the first diagnostic criteria for atypical hemolytic uremic syndrome was proposed and eculizumab was approved in Japan. The mean number of patients in each year before and after the approval of eculizumab was 4 and 18, respectively.

1. Omura T, Watanabe E, Otsuka Y, Yoshida Y, Kato H, Nangaku M, Miyata T, Oda S: Complete remission of thrombotic microangiopathy after treatment with eculizumab in a patient with non-Shiga toxin-associated bacterial enteritis: A case report. *Medicine,* 95**:** e4104, 2016

2. Kawaguchi K, Kawanishi K, Sato M, Itabashi M, Fujii A, Kanetsuna Y, Huchinoue S, Ohashi R, Koike J, Honda K, Nagashima Y, Nitta K: Atypical hemolytic uremic syndrome diagnosed four years after ABO-incompatible kidney transplantation. *Nephrology (Carlton),* 20 Suppl 2**:** 61-65, 2015

3. Matsukuma E, Imamura A, Iwata Y, Takeuchi T, Yoshida Y, Fujimura Y, Fan X, Miyata T, Kuwahara T: Postoperative atypical hemolytic uremic syndrome associated with complement c3 mutation. *Case Rep Nephrol,* 2014**:** 784943, 2014

4. Nagata A, Ohara A, Wakasugi D, Natori C, Ito S, Taguchi K, Fukami K, Okuda S: [A case of atypical hemolytic uremic syndrome successfully weaned from plasma exchange by treatment with eculizumab]. *Nihon Jinzo Gakkai Shi,* 56**:** 606-611, 2014

5. Ohta T, Urayama K, Tada Y, Furue T, Imai S, Matsubara K, Ono H, Sakano T, Jinno K, Yoshida Y, Miyata T, Fujimura Y: Eculizumab in the treatment of atypical hemolytic uremic syndrome in an infant leads to cessation of peritoneal dialysis and improvement of severe hypertension. *Pediatr Nephrol,* 30**:** 603-608, 2015

6. Okuda Y, Ishikura K, Terano C, Harada R, Hamada R, Hataya H, Ogata K, Honda M: Irreversible severe kidney injury and anuria in a 3-month-old girl with atypical haemolytic uraemic syndrome under administration of eculizumab. *Nephrology (Carlton),* 21**:** 261-265, 2016

7. Hisano M, Ashida A, Nakano E, Suehiro M, Yoshida Y, Matsumoto M, Miyata T, Fujimura Y, Hattori M: Autoimmune-type atypical hemolytic uremic syndrome treated with eculizumab as first-line therapy. *Pediatr Int,* 57**:** 313-317, 2015

8. Toyoda H, Wada H, Miyata T, Amano K, Kihira K, Iwamoto S, Hirayama M, Komada Y: Disease Recurrence After Early Discontinuation of Eculizumab in a Patient With Atypical Hemolytic Uremic Syndrome With Complement C3 I1157T Mutation. *Journal of pediatric hematology/oncology,* 38**:** e137-139, 2016

9. Terano C, Ishikura K, Hamada R, Yoshida Y, Kubota W, Okuda Y, Shinozuka S, Harada R, Iyoda S, Fujimura Y, Hamasaki Y, Hataya H, Honda M: Practical issues in using eculizumab for children with atypical hemolytic uremic syndrome in the acute phase: a review of 4 patients. *Nephrology (Carlton)*, 2017

10. Yamaguchi M, Hori M, Hiroshi N, Maruyama S: Postpartum atypical hemolytic uremic syndrome with complement factor H mutation complicated by reversible cerebrovascular constriction syndrome successfully treated with eculizumab. *Thrombosis research,* 151**:** 79-81, 2017

11. Yamamoto T, Watarai Y, Futamura K, Okada M, Tsujita M, Hiramitsu T, Goto N, Narumi S, Takeda A, Kobayashi T: Efficacy of Eculizumab Therapy for Atypical Hemolytic Uremic Syndrome Recurrence and Antibody-Mediated Rejection Progress After Renal Transplantation With Preformed Donor-Specific Antibodies: Case Report. *Transplantation proceedings,* 49**:** 159-162, 2017

12. Matsumoto T, Fan X, Ishikawa E, Ito M, Amano K, Toyoda H, Komada Y, Ohishi K, Katayama N, Yoshida Y, Matsumoto M, Fujimura Y, Ikejiri M, Wada H, Miyata T: Analysis of patients with atypical hemolytic uremic syndrome treated at the Mie University Hospital: concentration of C3 p.I1157T mutation. *Int J Hematol,* 100**:** 437-442, 2014

13. Yoshida Y, Miyata T, Matsumoto M, Shirotani-Ikejima H, Uchida Y, Ohyama Y, Kokubo T, Fujimura Y: A novel quantitative hemolytic assay coupled with restriction fragment length polymorphisms analysis enabled early diagnosis of atypical hemolytic uremic syndrome and identified unique predisposing mutations in Japan. *PLoS One,* 10**:** e0124655, 2015

14. Fan X, Yoshida Y, Honda S, Matsumoto M, Sawada Y, Hattori M, Hisanaga S, Hiwa R, Nakamura F, Tomomori M, Miyagawa S, Fujimaru R, Yamada H, Sawai T, Ikeda Y, Iwata N, Uemura O, Matsukuma E, Aizawa Y, Harada H, Wada H, Ishikawa E, Ashida A, Nangaku M, Miyata T, Fujimura Y: Analysis of genetic and predisposing factors in Japanese patients with atypical hemolytic uremic syndrome. *Mol Immunol,* 54**:** 238-246, 2013

15. Miyata T, Uchida Y, Ohta T, Urayama K, Yoshida Y, Fujimura Y: Atypical haemolytic uraemic syndrome in a Japanese patient with DGKE genetic mutations. In: *Thromb Haemost.*  Germany, 2015, pp 862-863

16. Edey MM: Thrombomodulin in atypical hemolytic-uremic syndrome. *N Engl J Med,* 361**:** 1511; author reply 1511, 2009
